# Dynamic Risk Measures for Anticipated Backward Doubly Stochastic Volterra Integral Equations

**DOI:** 10.3390/e23121580

**Published:** 2021-11-26

**Authors:** Liangliang Miao, Zhang Liu, Yijun Hu

**Affiliations:** 1 School of Mathematics and Statistics, Wuhan University, Wuhan 430072, China; yjhu.math@whu.edu.cn; 2 School of Computer and Information Engineering, Jiangxi Agricultural University, Nanchang 330045, China; liuzhang@whu.edu.cn

**Keywords:** dynamic risk measures, anticipated backward doubly stochastic Volterra integral equations, comparison theorems

## Abstract

Inspired by the consideration of some inside and future market information in financial market, a class of anticipated backward doubly stochastic Volterra integral equations (ABDSVIEs) are introduced to induce dynamic risk measures for risk quantification. The theory, including the existence, uniqueness and a comparison theorem for ABDSVIEs, is provided. Finally, dynamic convex risk measures by ABDSVIEs are discussed.

## 1. Introduction

It is well known that the concept of coherent risk measures with four axioms was first proposed to evaluate a risk position by [1], and further extended to convex risk measures by [2,3]. In addition to these static risk measures, a class of dynamic risk measures based on backward stochastic differential equations (BSDEs) has been widely studied.

The general theory of BSDEs, first established by [4], can be used to construct dynamic risk measures for risk evaluation in the field of finance and insurance. For instance, in a static and dynamic framework, Ref. [5] discussed a class of risk measures based on the theory of g-expectation of BSDEs, which was established by [6]. With the help of g-expectation of BSDEs, some equivalent characterizations of dynamic risk measures were provided in [7], and further developed by [8,9] to dynamic risk measures for processes. Ref. [10] extended dynamic risk measures induced by the g-expectation of BSDEs to the multidimensional case. Recently, Ref. [11] studied dynamic risk measures related to BSDEs with jumps under an enlargement of filtration, and presented a numerical approach for them. Moreover, under the framework of set-valued BSDEs, Ref. [12] introduced and studied set-valued risk measures.

Another kind of dynamic risk measures based on backward stochastic Volterra integral equations (BSVIEs) are worth exploring and studying. BSVIEs, as a generalized form of BSDEs, were initially considered to induce dynamic risk measures in [13], where the concept of M-solution was introduced to solve the problem of uniqueness to BSVIEs. Ref. [14] stated that dynamic risk measures induced by BSVIEs are time-inconsistent. The time-inconsistent dynamic risk measures for BSVIEs with jumps were considered by [15,16]. Ref. [17] discussed equilibrium dynamic risk measures induced by quadratic BSVIEs and explored equilibrium recursive utility processes.

Dynamic risk measures based on the classical BSDEs and BSVIEs have their own advantages. Compared with risk measures by the classical BSDEs, risk measures by BSVIEs allow the terminal objective of a wealth process (which is commonly described by a random variable in the classical BSDEs seting) to be described by a stochastic process (which only needs to be measurable to the information at terminal time). Risk measures by BSVIEs also consider the time value of a wealth process. Unfortunately, risk measures by BSVIEs are time-inconsistent, not time-consistent, while risk measures based on the classical BSDEs are time-consistent in a sense.

In recent years, BSVIEs have been widely studied by many other researchers. For example, Ref. [18] proved the existence and uniqueness of BSVIEs with jumps in Hilbert spaces. The unique solvability of BSVIEs under more general stochastic non-Lipschitz conditions was shown in [19]. Furthermore, Ref. [20] provided some theoretical research on backward doubly stochastic Volterra integral equations (BDSVIEs), including the well-posedness of solutions, a comparison theorem, and a related optimization problem. The similar work for anticipated BSVIEs, compared with [20], was presented in [21]. Refs. [22,23] considered extended BSVIEs and some related control problems.

In this work, we introduce the following Volterra integral equation to simulate a wealth process,

(1) $$\left\{\begin{array}{cc} Y(t) & =\xi \left(t\right)\;+\;\int_{t}^{T}f\left(t,s,Y(s),Z(t,s),Z(s,t),Y(s+\delta (s)),Z(t,s+\gamma (s)),\right.\\ \; & \;\;\;\;\left.Z(s+\gamma (s),t)\right)ds+\int_{t}^{T}g\left(t,s,Y\left(s\right),Z\left(t,s\right),Z\left(s,t\right)\right)d\overset{\leftarrow }{B}\left(s\right)\\ \; & \;\;\;\;-\int_{t}^{T}Z\left(t,s\right)dW\left(s\right),\;\;\;t\in \left[0,T\right];\\ Y(t) & =\xi (t),\;\;\;t\in [T,T+K];\\ Z(t,s) & =\eta \left(t,s\right),\;\;\;\left(t,s\right)\in {\left[0,T+K\right]}^{2}\backslash {\left[0,T\right]}^{2}. \end{array}\right.$$

Here, ξ is a given $R^{n}$-valued stochastic process, $f\left(\cdot \right):{\left[0,T\right]}^{2}\times R^{n}\times R^{n\times d}\times R^{n\times d}\times R^{n}\times R^{n\times d}\times R^{n\times d}\to R^{n}$ and $g\left(\cdot \right):{\left[0,T\right]}^{2}\times R^{n}\times R^{n\times d}\times R^{n\times d}\to R^{n}$ satisfy some given conditions, and ζ(·) and δ(·) are some given $R^{+}$-valued continuous functions satisfying s+ζ(s)≤T+K,s+δ(s)≤T+K,s∈[0,T]. The above equation is called anticipated backward doubly stochastic Volterra integral equation (ABDSVIE). ξ is called the terminal condition (or sometimes the free term), and f(·) and g(·) are called the generator or the driver of (1). ABDSVIEs can be seen as a generalized form of BSVIEs and BDSVIEs. ABDSVIEs can also be seen as a generalized form of the class of Volterra integral equations (VIEs) of the first kind, which belong to the class of so-called weakly regular integral equations. In [24], this monograph systematically provided the detailed related theories and methods, including VIEs of the first/second kind, the control and optimization problem, and the various applications of Volterra models. In [25], they applied some special non-linear VIEs to an optimal control of a multifactor vintage capital model. To the best of our knowledge, no study about ABDSVIEs is available up to now.

Now, we interpret the motivation for introducing ABDSVIEs. Some inside and future market information is considered in the financial market. First, there may be some inside information, which can be only observed by some specific investors in the derivatives market. This inside information decreases as the trading day approaches. In other words, this inside information will be observed by all investors on the trading day. Therefore, this inside information, which may not be observed in practice, can be simulated by a Brownian motion *B* with a backward Itô integral. Second, because these specific investors have more market information than ordinary investors, the wealth process *Y* can be predicted by these specific investors for a short period of time in the future. This shows that the generator contains not only the current market information, but also the future information. Based on the above idea, we introduce ABDSVIEs (1) to derive dynamic risk measures for the risk quantification of the wealth process of some investors.

In this work, inspired by the consideration of some inside and future market information in the financial market, a class of ABDSVIEs are introduced and used to induce dynamic risk measures for risk quantification. The theory, including the existence, uniqueness and a comparison theorem for ABDSVIEs, is provided. Finally, dynamic risk measures by ABDSVIEs are presented. The theory of ABDSVIEs extends the model and results of [20,21].

This paper is organized as follows. Section 2 provides some preliminaries including the definition of M-solution, and some results on BDSVIEs. Section 3 contains our main results; that is, the theory for ABDSVIEs including the existence, uniqueness, a comparison theorem, and their applications in risk measures are presented. All proofs of the main results of this paper are addressed in Section 4. Finally, conclusions are summarized.

## 2. Preliminaries

Throughout this paper, let (Ω,F,P) be a probability space. Let {W(t),0≤t<∞} be a *d*-dimensional standard Brownian motion, and let {B(t),0≤t<∞} is a *l*-dimensional standard Brownian motion. {W(t),0≤t<∞} and {B(t),0≤t<∞} are mutually independent. Let T>0 denote a given terminal time. Suppose that ▵ and ${▵}^{c}$ are defined as follows.

$$▵:=\left\{\left(t,s\right)\in {\left[0,T\right]}^{2}\;|\;t\leq s\right\}\;\;\;\;\mathrm{and}\;\;\;\;{▵}^{c}:=\left\{\left(t,s\right)\in {\left[0,T\right]}^{2}\;|\;t>s\right\}.$$

Let the class of P-null sets of F be denoted by N. Let $F=\{F_{t},t\in \left[0,T\right]\}$ be defined by

(2) $$F_{t}:=F_{t}^{W}\vee F_{t,T}^{B},$$

where $F_{s,t}^{\vartheta }:=\sigma \left\{\vartheta \left(u\right)-\vartheta \left(s\right);s\leq u\leq t\right\}\vee N$ and $F_{t}^{\vartheta }:=F_{0,t}^{\vartheta }$ (the process {ϑ(·)} represents {W(t),0≤t<∞} or {B(t),0≤t<∞}). Let H represent Euclidean space. |x| denotes its Euclidean norm for any $x\in R^{n}$, and let |A| be defined by $\left|A\right|=\sqrt{TrAA^{*}}$ for $A\in R^{n\times d}$. We introduce the following spaces.

- $L_{F_{t}}^{2}\left(\Omega ;H\right)$ denotes the set of $F_{t}$-measurable random variables ξ:Ω→H satisfying $E[|\xi {|}^{2}]<\infty$.
- $L_{F_{T}}^{2}\left(0,T+K;H\right)$ denotes the set of $F_{T\vee t}$-measurable processes ξ:Ω×[0,T+K]→H satisfying
  $$E\left[\int_{0}^{T+K}{\left|\xi \left(t\right)\right|}^{2}dt\right]<\infty .$$
- $L_{F}^{2}\left(0,T;H\right)$ denotes the set of F-measurable processes h:Ω×[0,T]→H satisfying
  $$E\left[\int_{0}^{T}{\left|h\left(t\right)\right|}^{2}dt\right]<\infty .$$
- $L_{F}^{2}\left(▵;H\right)$ denotes the set of F-measurable processes Z:Ω×▵→H satisfying that s→Z(t,s) is F-measurable on [t,T] with
  $$E\left[\int_{0}^{T}\int_{t}^{T}{\left|Z\left(t,s\right)\right|}^{2}dsdt\right]<\infty .$$
- $L_{F}^{2}\left({\left[0,T\right]}^{2};H\right)$ denotes the set of F-measurable processes $Z:\Omega \times {\left[0,T\right]}^{2}\to H$ satisfying that s→Z(t,s) is F-measurable on [0,T] with
  $$E\left[\int_{0}^{T}\int_{0}^{T}{\left|Z\left(t,s\right)\right|}^{2}dsdt\right]<\infty .$$

$$H_{▵}^{2}\left[0,T\right]:=L_{F}^{2}\left(0,T;H\right)\times L_{F}^{2}\left(▵;H\right),\;\;\;H^{2}\left[0,T\right]:=L_{F}^{2}\left(0,T;H\right)\times L_{F}^{2}\left({\left[0,T\right]}^{2};H\right).$$

Notice that $H_{▵}^{2}\left[0,T\right]$ and $H^{2}\left[0,T\right]$ are a Hilbert space under the following norms

$${∥(Y,Z,K)∥}_{H_{▵}^{2}\left[0,T\right]}^{2}:=E\left[\int_{0}^{T}\left(e^{\beta t}{\left|Y\left(t\right)\right|}^{2}+\int_{t}^{T}e^{\beta s}{\left|Z\left(t,s\right)\right|}^{2}ds\right)dt\right],$$

and

$${∥(Y,Z,K)∥}_{H^{2}\left[0,T\right]}^{2}:=E\left[\int_{0}^{T}\left(e^{\beta t}{\left|Y\left(t\right)\right|}^{2}+\int_{0}^{T}e^{\beta s}{\left|Z\left(t,s\right)\right|}^{2}ds\right)dt\right],$$

respectively. Similarly, we can define $L_{F_{T}}^{2}\left(0,T;H\right),L_{F}^{2}\left(0,T+K;H\right),L_{F}^{2}\left({\left[0,T+K\right]}^{2};H\right)$, $H_{▵}^{2}\left[0,T+K\right],H^{2}\left[0,T+K\right]$, etc.

Consider the following equtions:

(3) $$\begin{array}{cc} Y(t)= & \xi \left(t\right)+\int_{t}^{T}f\left(t,s,Y(s),Z(t,s),Z(s,t)\right)ds\\ \; & +\int_{t}^{T}g\left(t,s,Y\left(s\right),Z\left(t,s\right),Z\left(s,t\right)\right)d\overset{\leftarrow }{B}\left(s\right)\\ \; & -\int_{t}^{T}Z\left(t,s\right)dW\left(s\right),\;\;\;t\in \left[0,T\right], \end{array}$$

where (ξ(·),f(·),g(·)) satisfies the following assumptions.

**Assumption** **1.**

*(i)*  $\xi \left(\cdot \right)\in L_{F_{T}}^{2}\left(0,T;R^{n}\right)$.
*(ii)*  $f:\Omega \times ▵\times R^{n}\times R^{n\times d}\times R^{n\times d}\to R^{n}$*and*$g:\Omega \times ▵\times R^{n}\times R^{n\times d}\times R^{n\times d}\to R^{n\times l}$*are jointly measurable for any*$\left(y,z,\varsigma \right)\in R^{n}\times R^{n\times d}\times R^{n\times d}$*such that*
  - $f\left(\cdot ,\cdot ,y,z,\varsigma \right)\in L_{F}^{2}\left(▵;R^{n}\right),\;\;\;\;g\left(\cdot ,\cdot ,y,z,\varsigma \right)\in L_{F}^{2}\left(▵;R^{n\times l}\right).$
  - *There exists a L>0 and $0<\alpha <\frac{1}{T+2}$ such that for any $y_{1},y_{2}\in R^{n},z_{1},z_{2},\varsigma_{1},\varsigma_{2}\in R^{n\times d}$,*
    $$\begin{array}{cc} & {\left|f\left(t,s,y_{1},z_{1},\varsigma_{1}\right)-f\left(t,s,y_{2},z_{2},\varsigma_{2}\right)\right|}^{2}\leq L\left(|y_{1}-y_{2}{|}^{2}+|z_{1}-z_{2}{|}^{2}+{\left|\varsigma_{1}-\varsigma_{2}\right|}^{2}\right),\\ & {\left|g\left(t,s,y_{1},z_{1},\varsigma_{1}\right)-g\left(t,s,y_{2},z_{2},\varsigma_{2}\right)\right|}^{2}\leq \alpha \left(|y_{1}-y_{2}{|}^{2}+|z_{1}-z_{2}{|}^{2}+{\left|\varsigma_{1}-\varsigma_{2}\right|}^{2}\right). \end{array}$$

**Remark** **1.** *Note that $F=\{F_{t},t\in \left[0,T\right]\}$ in (2) is not a filtration. In [20], based on the idea of M-solution to BSVIEs, the authors defined a filtration $G=\{G_{t},t\in \left[0,T\right]\}$ by $G_{t}:=F_{t}^{W}\vee F_{0,T}^{B}$ to introduce the M-solution to BDSVIEs.*

We now introduce the definition of M-solution to BDSVIE (3) and some results concerning BDSVIEs. In detail, the following Propositions 1–3 are Theorem 3.3, Lemma 2.5 and Theorem 4.2 of [20], respectively.

**Definition** **1.** *A pair of processes $\left(Y\left(\cdot \right),Z\left(\cdot ,\cdot \right)\right)\in H^{2}\left[0,T\right]$ is called a solution of BDSVIE (3) if it satisfies (3) in the usual Itô’s sense for Lebesgue measure almost every t∈[0,T]. Moreover, a solution (Y(·),Z(·,·)) is called an M-solution of BDSVIE (3) if for any S∈[0,T), the following relation holds:*

(4) $$\begin{array}{c} Y\left(t\right)=E\left[Y\left(t\right)|F_{S}\right]+\int_{S}^{t}Z\left(t,s\right)dB\left(s\right),\;\;\;\;\;a.e.t\in \left[S,T\right] \end{array}$$

**Proposition** **1.** *Under Assumption (H1), there has a unique M-solution $\left(Y\left(\cdot \right),Z\left(\cdot ,\cdot \right)\right)\in H^{2}\left[0,T\right]$ of BDSVIE (3).*

**Proposition** **2.** *Let $f\left(\cdot \right)\in L_{F}^{2}\left(▵;R^{n}\right)$ and $g\left(\cdot \right)\in L_{F}^{2}\left(▵;R^{n\times l}\right)$. Then for any $\xi \left(\cdot \right)\in L_{F_{T}}^{2}$$\left(0,T;R^{n}\right)$, BDSVIE:*

(5) $$\begin{array}{cc} Y(t)= & \xi \left(t\right)+\int_{t}^{T}f\left(t,s\right)ds+\int_{t}^{T}g\left(t,s\right)d\overset{\leftarrow }{B}\left(s\right)\\ \; & -\int_{t}^{T}Z\left(t,s\right)dW\left(s\right),\;\;\;t\in \left[0,T\right], \end{array}$$

*has an unique solution $\left(Y\left(\cdot \right),Z\left(\cdot ,\cdot \right)\right)\in H_{▵}^{2}\left[0,T\right]$. Moreover, the following estimate holds for for some constant β>0,*

(6) $$\begin{array}{cc} \; & E\left[\int_{0}^{T}\left(e^{\beta t}{\left|Y\left(t\right)\right|}^{2}+\int_{t}^{T}e^{\beta s}{\left|Z\left(t,s\right)\right|}^{2}ds\right)dt\right]\\ \; & \leq Ce^{\beta T}E\left[\int_{0}^{T}{\left|\xi \left(t\right)\right|}^{2}dt\right]+\frac{C}{\beta }E\left[\int_{0}^{T}\int_{t}^{T}e^{\beta s}{\left|f\left(t,s\right)\right|}^{2}dsdt\right]\\ \; & \;\;\;\;+E\left[\int_{0}^{T}e^{\beta t}\int_{t}^{T}{\left|g\left(t,s\right)\right|}^{2}dsdt\right]+E\left[\int_{0}^{T}\int_{t}^{T}e^{\beta s}{\left|g\left(t,s\right)\right|}^{2}dsdt\right]. \end{array}$$

**Proposition** **3.** *For i=1,2, suppose that $f^{i}:\Omega \times ▵\times R\times R\to R$ and g:Ω×▵×R×R→R satisfy Assumption (H1). For any given $\xi^{1}\left(\cdot \right),\xi^{2}\left(\cdot \right)\in L_{F_{T}}^{2}\left(0,T;R\right)$, let $\left(Y^{i}\left(\cdot \right),Z^{i}\left(\cdot ,\cdot \right)\right)$ denote the solution of*

(7) $$\begin{array}{cc} Y^{i}\left(t\right)= & \xi^{i}\left(t\right)+\int_{t}^{T}f^{i}\left(t,s,Y^{i}\left(s\right),Z^{i}\left(t,s\right)\right)ds\\ \; & +\int_{t}^{T}g\left(t,s,Y^{i}\left(s\right),Z^{i}\left(t,s\right)\right)d\overset{\leftarrow }{B}\left(s\right)-\int_{t}^{T}Z^{i}\left(t,s\right)dB\left(s\right),\;\;\;t\in \left[0,T\right]. \end{array}$$

*Suppose that for any (t,y,z)∈[0,s]×R×R,*

$$f^{1}\left(t,s,y,z\right)\leq \bar{f}\left(t,s,y,z\right)\leq f^{2}\left(t,s,y,z\right)\;\;\;\;a.s.,\;a.e.\;s\in \left[0,T\right].$$

*The generator $\bar{f}:\Omega \times ▵\times R\times R\to R$ also satisfies Assumption (H1) and $y\to \bar{f}\left(t,s,y,z\right)$ is nondecreasing. If $\xi^{1}\left(t\right)\leq \xi^{2}\left(t\right)$, a.s. t∈[0,T], then*

$$Y^{1}\left(t\right)\leq Y^{2}\left(t\right),\;\;\;\;a.s.,\;\;t\in \left[0,T\right].$$

For the convenience of readers, we rewrite the ABDSVIE (1) as follows:

(8) $$\left\{\begin{array}{cc} Y(t) & =\xi \left(t\right)\;+\;\int_{t}^{T}f\left(t,s,Y(s),Z(t,s),Z(s,t),Y(s+\delta (s)),Z(t,s+\gamma (s)),\right.\\ \; & \;\;\;\;\left.Z(s+\gamma (s),t)\right)ds+\int_{t}^{T}g\left(t,s,Y\left(s\right),Z\left(t,s\right),Z\left(s,t\right)\right)d\overset{\leftarrow }{B}\left(s\right)\\ \; & \;\;\;\;-\int_{t}^{T}Z\left(t,s\right)dW\left(s\right),\;\;\;t\in \left[0,T\right];\\ Y(t) & =\xi (t),\;\;\;t\in [T,T+K];\\ Z(t,s) & =\eta \left(t,s\right),\;\;\;\left(t,s\right)\in {\left[0,T+K\right]}^{2}\backslash {\left[0,T\right]}^{2}. \end{array}\right.$$

where ζ(·) and δ(·) are some given nonnegative continuous functions satisfying the following conditions.

(i) K≥0 is a constant with
  s+ζ(s)≤T+K,s+δ(s)≤T+K,s∈[0,T].
(ii) For any t∈[0,T] and any nonnegative integrable functions $f_{1}\left(\cdot \right)$ and $f_{2}\left(\cdot \right)$, there has a constant Γ≥0 with
  (9) $$\begin{array}{cc} \; & \int_{t}^{T}f_{1}\left(s+\zeta \left(s\right)\right)ds\leq \Gamma \int_{t}^{T+K}f_{1}\left(s\right)ds,\\ \; & \int_{t}^{T}f_{2}\left(t,s+\delta \left(s\right)\right)ds\leq \Gamma \int_{t}^{T+K}f_{2}\left(t,s\right)ds,\\ \; & \int_{t}^{T}f_{2}\left(s+\delta \left(s\right),t\right)ds\leq \Gamma \int_{t}^{T+K}f_{2}\left(s,t\right)ds. \end{array}$$

**Remark** **2.** *ABDSVIEs (8) are a generalized form of BDSVIE (3). The definition of M-solution to BDSVIE (3) is applicable to ABDSVIEs (8) in fact.*

**Assumption** **2.**

*(i)*  $\xi \left(\cdot \right)\in L_{F_{T}}^{2}\left(0,T;R^{n}\right)$.
*(ii)*  $f\left(t,s,y,z,\varsigma ,\psi ,\varphi ,\phi \right):\Omega \times ▵\times R^{n}\times R^{n\times d}\times R^{n\times d}\times L_{F_{u_{1}}}^{2}\left(\Omega ;R^{n}\right)\times L_{F_{u_{2}}}^{2}\left(\Omega ;R^{n\times d}\right)\times L_{F_{u_{3}}}^{2}\left(\Omega ;R^{n\times d}\right)\to L_{F_{s}}^{2}\left(\Omega ;R^{n}\right)$, *where $u_{1},u_{2},u_{3}\in \left[s,T+K\right]$, and $g:\Omega \times ▵\times R^{n}\times R^{n\times d}\times R^{n\times d}\to R^{n\times l}$ are jointly measurable such that*

- $f\left(\cdot ,\cdot ,y,z,\varsigma ,\psi ,\varphi ,\phi \right)\in L_{F}^{2}\left(▵;R^{n}\right),\;\;\;\;g\left(\cdot ,\cdot ,y,z,\varsigma \right)\in L_{F}^{2}\left(▵;R^{n\times l}\right).$
- *There has a*L>0 and $0<\alpha <\frac{1}{T+2}$ satisfying for any $y_{1},y_{2}\in R^{n},z_{1},z_{2},\varsigma_{1},\varsigma_{2}\in R^{n\times d},\psi_{1}\left(\cdot \right),\psi_{2}\left(\cdot \right)\in L_{F}^{2}\left(s,T+K;R^{n}\right),\varphi_{1}\left(\cdot \right),\varphi_{2}\left(\cdot \right),$
  $\phi_{1}\left(\cdot \right),\phi_{2}\left(\cdot \right)\in L_{F}^{2}\left(s,T+K;R^{n\times d}\right)$*,*
  $$\begin{array}{cc} & \left|f(t,s,y_{1},z_{1},\varsigma_{1},\psi_{1}\left(u_{1}\right),\varphi_{1}\left(t,u_{2}\right),\phi_{1}\left(u_{3},t\right))\right.\\ & \;\;\;\;{\left.-f(t,s,y_{2},z_{2},\varsigma_{2},\psi_{2}\left(u_{1}\right),\varphi_{2}\left(t,u_{2}\right),\phi_{2}\left(u_{3},t\right))\right|}^{2}\\ & \leq L(|y_{1}\;-\;y_{2}{|}^{2}+|z_{1}\;-\;z_{2}{|}^{2}\;+\;{\left|\varsigma_{1}\;-\;\varsigma_{2}\right|}^{2}+\;E\left[|\psi_{1}\left(u_{1}\right)\;-\;\psi_{2}\left(u_{1}\right){|}^{2}\;\right.\\ & \;\;\;\;\left.+\;|\varphi_{1}\left(t,u_{2}\right)\;-\;\varphi_{2}\left(t,u_{2}\right){|}^{2}\;+\;|\phi_{1}\left(u_{3},t\right)\;-\;\phi_{2}\left(u_{3},t\right){|}^{2}|F_{s}\right]),\\ & {\left|g\left(t,s,y_{1},z_{1},\varsigma_{1}\right)-g\left(t,s,y_{2},z_{2},\varsigma_{2}\right)\right|}^{2}\\ & \leq \alpha \left(|y_{1}-y_{2}{|}^{2}+|z_{1}-z_{2}{|}^{2}+{\left|\varsigma_{1}-\varsigma_{2}\right|}^{2}\right). \end{array}$$

Now, we define a class of dynamic risk measures for ABDSVIEs. By slightly modifying the definition of dynamic risk measures in [13], the terminal time of dynamic risk measures can be extended to the time T+K. The detail definition of dynamic risk measures is as follows.

**Definition** **2.** *A map $\rho :\left[0,T+K\right]\times L_{F_{T}}^{2}\left(0,T+K;R\right)\to L_{F}^{2}\left(0,T+K;R\right)$ is called a dynamic risk measure if ρ satisfies the following conditions:*

- *(Monotonicity) For each $\xi \left(\cdot \right),\eta \left(\cdot \right)\in L_{F_{T}}^{2}\left(0,T+K;R\right)$, if*
  ξ(s)≤η(s),a.s.,s∈[t,T+K],
    *for some t∈[0,T+K), then*
  ρ(s;ξ(·))≥ρ(s;η(·)),a.s.,s∈[t,T+K].
- *(Cash invariance) There has a deterministic integrable function r(·) satisfying for each $\xi \left(\cdot \right)\in L_{F_{T}}^{2}\left(\left[0,T+K\right];R\right)$ and for each constant c,*
  $$\rho \left(t;\xi \left(\cdot \right)+c\right)=\rho \left(t;\xi \left(\cdot \right)\right)-ce^{-\int_{t}^{T}r\left(s\right)ds},\;\;\;\;a.s.,\;t\in \left[0,T+K\right].$$
- *(Past independence) For each $\xi \left(\cdot \right),\eta \left(\cdot \right)\in L_{F_{T}}^{2}\left(0,T+K;R\right)$, if*
  ξ(s)=η(s),a.s.,s∈[t,T+K]
    *for some t∈[0,T+K), then*
  ρ(t;ξ(·))=ρ(t;η(·)),a.s.

*Moreover, the dynamic risk measure ρ is called a dynamic coherent risk measure if ρ satisfies the following conditions:*

- *(Subadditivity) ρ(t;ξ(·)+η(·))≤ρ(t;ξ(·))+ρ(t;η(·)),a.s.,t∈[0,T+K].*
- *(Positive homogeneity) For each $\xi \left(\cdot \right)\in L_{F_{T}}^{2}\left(0,T+K;R\right)$ and for each λ>0,*
  ρ(t;λξ(·))=λρ(t;ξ(·)),a.s.,t∈[0,T+K].
  *and the dynamic risk measure ρ is called a dynamic convex risk measure if ρ satisfies the following condition:*
- *(Convexity) For each $\xi \left(\cdot \right),\eta \left(\cdot \right)\in L_{F_{T}}^{2}\left(0,T+K;R\right)$ and for each λ∈[0,1],*
  $$\rho \left(t;\lambda \xi (\cdot )+(1-\lambda )\eta (\cdot )\right)\leq \lambda \rho \left(t;\xi \left(\cdot \right)\right)+\left(1-\lambda \right)\rho \left(t;\eta \left(\cdot \right)\right),\;\;\;\;a.s.,\;t\in \left[0,T+K\right].$$

## 3. Main Results

Now, we present some main results concerning ABDSVIEs. Namely, we first present the well-posedness of ABDSVIEs, and then a comparison theorem for ABDSVIEs will be given. Finally, dynamic convex risk measures via ABDSVIEs will be discussed.

Let $M^{2}\left[0,T+K\right]$ denote the space of all pairs $\left(Y\left(\cdot \right),Z\left(\cdot ,\cdot \right)\right)\in H^{2}\left[0,T+K\right]$ with for any S∈[0,T),

(10) $$Y\left(t\right)=E\left[Y\left(t\right)\right]+\int_{S}^{t}Z\left(t,s\right)dW\left(s\right),\;\;\;\;\;t\in \left[0,T+K\right].$$

Let ${∥\cdot ∥}_{M^{2}\left[0,T+K\right]}^{2}$ be defined by

(11) $$\begin{array}{cc} \; & {∥\left(Y(\cdot ),Z(\cdot ,\cdot )\right)∥}_{M^{2}\left[0,T+K\right]}^{2}\\ \; & :=E\left[\int_{0}^{T+K}\left(e^{\beta t}{\left|Y\left(t\right)\right|}^{2}+\int_{t}^{T+K}e^{\beta s}{\left|Z\left(t,s\right)\right|}^{2}ds\right)dt\right]. \end{array}$$

By (10), we get for any $\left(Y\left(\cdot \right),Z\left(\cdot ,\cdot \right)\right)\in H^{2}\left[0,T+K\right]$

$$\begin{array}{cc} & E\left[\int_{0}^{T+K}e^{\beta t}{\left|Y\left(t\right)\right|}^{2}dt+\int_{0}^{T+K}\int_{t}^{T+K}e^{\beta s}{\left|Z\left(t,s\right)\right|}^{2}dsdt\right]\\ & \leq E\left[\int_{0}^{T+K}e^{\beta t}{\left|Y\left(t\right)\right|}^{2}dt+\int_{0}^{T+K}\int_{t}^{T+K}e^{\beta s}{\left|Z\left(t,s\right)\right|}^{2}dsdt\right]\\ & \;\;\;\;+E\left[\int_{0}^{T+K}\int_{0}^{t}e^{\beta s}{\left|Z\left(t,s\right)\right|}^{2}dsdt\right]\\ & \leq 2E\left[\int_{0}^{T+K}\left(e^{\beta t}{\left|Y\left(t\right)\right|}^{2}+\int_{t}^{T+K}e^{\beta s}{\left|Z\left(t,s\right)\right|}^{2}ds\right)dt\right], \end{array}$$

which can imply that

$$\begin{array}{cc} {∥\left(Y(\cdot ),Z(\cdot ,\cdot )\right)∥}_{M^{2}\left[0,T+K\right]}^{2} & \leq {∥\left(Y(\cdot ),Z(\cdot ,\cdot )\right)∥}_{H^{2}\left[0,T+K\right]}^{2}\\ & \leq 2{∥\left(Y(\cdot ),Z(\cdot ,\cdot )\right)∥}_{M^{2}\left[0,T+K\right]}^{2}. \end{array}$$

Thus, from the above inequality, the norms ${∥\cdot ∥}_{M^{2}\left[0,T+K\right]}^{2}$ and ${∥\cdot ∥}_{H^{2}\left[0,T+K\right]}^{2}$ are equivalent in $M^{2}\left[0,T+K\right]$. The first main result is concerning on the theory of ABDSVIEs.

**Theorem** **1.** *Suppose that Assumption (H2) holds. Let $\left(\xi \left(\cdot \right),\eta \left(\cdot ,\cdot \right)\right)\in M^{2}\left[0,T+K\right]$, then ABDSVIE (8) has a unique M-solution $\left(Y\left(\cdot \right),Z\left(\cdot ,\cdot \right)\right)\in M^{2}\left[0,T+K\right]$.*

Note that, in ABDSVIE (8), when $f\left(\cdot \right)=f\left(t,s,Y(s),Z(t,s),Y(s+\zeta (s)),\right.$$\left.Z(t,s+\delta (s))\right)$ and g(·)=g(t,s,Y(s),Z(t,s)), the notation of M-solution is not necessary in fact.

Consider the following ABDSVIE:

(12) $$\left\{\begin{array}{cc} Y(t) & =\xi \left(t\right)+\int_{t}^{T}f\left(t,s,Y(s),Z(t,s),Y(s+\zeta (s)),Z(t,s+\delta (s))\right)ds\\ \; & \;\;\;\;+\int_{t}^{T}g\left(t,s,Y\left(s\right),Z\left(t,s\right)\right)d\overset{\leftarrow }{B}\left(s\right)\\ \; & \;\;\;\;-\int_{t}^{T}Z\left(t,s\right)dW\left(s\right),\;\;\;t\in \left[0,T\right];\\ Y(t) & =\xi (t),\;\;\;t\in [T,T+K];\\ Z(t,s) & =\eta \left(t,s\right),\;\;\;\left(t,s\right)\in {\left[0,T+K\right]}^{2}\backslash {\left[0,T\right]}^{2},t\leq s. \end{array}\right.$$

**Corollary** **1.** *Suppose that Assumption (H2) holds. Let $\left(\xi \left(\cdot \right),\eta \left(\cdot ,\cdot \right)\right)\in H_{▵}^{2}\left[0,T+K\right]$, then ABDSVIE (12) has a unique solution $\left(Y\left(\cdot \right),Z\left(\cdot ,\cdot \right)\right)\in H_{▵}^{2}\left[0,T+K\right]$.*

**Remark** **3.** *For t∈[0,T+K], ξ(t) is not $F_{T\vee t}$-measurable, but $F_{t}$-adapted in Theorem 1 and Corollary 1. In fact, we suppose that ξ(t) is $F_{t}$-adapted just for simplicity of presentation, and the results of Theorem 1 and Corollary 1 remain true when ξ(t) is $F_{T\vee t}$-measurable.*

Furthermore, the second main result concerning a comparison theorem for ABDSVIEs will be presented in a one-dimensional setting.

**Theorem** **2.** *For i=1,2, suppose that $\xi^{i}\left(\cdot \right),f^{i}\left(\cdot \right)$ and g(·) satisfy Assumption (H2). Let the solution to ABDSVIE (13) be denoted by $\left(Y^{i}\left(\cdot \right),Z^{i}\left(\cdot ,\cdot \right)\right)$,*

(13) $$\left\{\begin{array}{cc} Y^{i}\left(t\right) & =\xi^{i}\left(t\right)+\int_{t}^{T}f^{i}\left(t,s,Y^{i}\left(s\right),Z^{i}\left(t,s\right),Y^{i}\left(s+\zeta \left(s\right)\right)\right)ds\\ \; & \;\;\;\;+\int_{t}^{T}g\left(t,s,Y^{i}\left(s\right),Z^{i}\left(t,s\right)\right)d\overset{\leftarrow }{B}\left(s\right)\\ \; & \;\;\;\;-\int_{t}^{T}Z^{i}\left(t,s\right)dW\left(s\right),\;\;\;t\in \left[0,T\right];\\ Y^{i}\left(t\right) & =\xi^{i}\left(t\right),\;\;\;t\in \left[T,T+K\right]. \end{array}\right.$$

*Suppose that for each $\left(t,y,z,\psi \right)\in \left[0,s\right]\times R\times R\times L_{F_{u}}^{2}\left(\Omega ;R\right)$, u∈[s,T+K],*

$$f^{1}\left(t,s,y,z,\psi \right)\leq \bar{f}\left(t,s,y,z,\psi \right)\leq f^{2}\left(t,s,y,z,\psi \right)\;\;\;\;a.s.,\;a.e.\;s\in \left[0,T\right],$$

*The generator $\bar{f}:\Omega \times ▵\times R\times R\times L_{F_{u}}^{2}\left(\Omega ;R\right)\to R$, u∈[s,T+K], also satisfies Assumption (H2), $\bar{f}\left(t,s,y,z,\psi \right)$ is nondecreasing in y, i.e., $f\left(t,s,y_{1},z,\psi \right)\leq f\left(t,s,y_{2},z,\psi \right)$ if $y_{1}\leq y_{2}$, and $\bar{f}\left(t,s,y,z,\psi \right)$ is increasing with respect to ψ, i.e., $\bar{f}\left(t,s,y,z,\psi_{1}\left(u\right)\right)\leq \bar{f}\left(t,s,y,z,\psi_{2}\left(u\right)\right)$ if $\psi_{1}\left(u\right)\leq \psi_{2}\left(u\right)$ with $\psi_{1}\left(u\right),\psi_{2}\left(u\right)\in L_{F}^{2}\left(s,T+K;R\right)$. If $\xi^{1}\left(t\right)\leq \xi^{2}\left(t\right)$, a.s. t∈[0,T+K], then we have*

$$Y^{1}\left(t\right)\leq Y^{2}\left(t\right),\;\;\;\;a.s.,\;\;t\in \left[0,T+K\right].$$

Finally, as an application of ABDSVIEs, under Assumption (H2), we shall induce risk measures by ABDSVIEs defined by

(14) $$\begin{array}{c} \rho_{t}\left(\xi \left(\cdot \right)\right)=Y\left(t\right),\;\;\;\;\;\forall t\in \left[0,T+K\right], \end{array}$$

where Y(·) is the solution (Y(·),Z(·,·)) to anticipated BDSVIEs (15),

(15) $$\left\{\begin{array}{cc} Y(t) & =-\xi \left(t\right)+\int_{t}^{T}f\left(t,s,Y(s),Z(t,s),Y(s+\zeta (s))\right)ds\\ \; & \;\;\;\;+\int_{t}^{T}g\left(t,s,Z\left(t,s\right)\right)d\overset{\leftarrow }{B}\left(s\right)-\int_{t}^{T}Z\left(t,s\right)dW\left(s\right),\;\;\;t\in \left[0,T\right];\\ Y(t) & =-\xi (t),\;\;\;t\in [T,T+K]. \end{array}\right.$$

Let $f\left(t,s,y,z,\psi \right):\Omega \times ▵\times R\times R\times L_{F_{u}}^{2}\left(\Omega ;R\right)\to R,u\in \left[s,T+K\right]$ be definedas follows,

(16) $$\begin{array}{c} f\left(t,s,y,z,\psi \right)=r\left(s\right)\left(y+\psi \right)+f_{0}\left(t,s,z\right), \end{array}$$

where r(·) is $R^{+}$-valued deterministic function.

Now, we state dynamic risk measures via ABDSVIEs and give the following main theorem.

**Theorem** **3.** *Suppose that the generator f, given by (16), and the generator g satisfy Assumption (H2). Then ρ, defined by (14), is a dynamic convex risk measure if the following conditions hold:*

*(i)*  *(Convexity) Suppose that f is convex in*(y,z,ψ)*, i.e., for each*$\left(y_{1},z_{1},\psi_{1}\right),$
  $\left(y_{2},z_{2},\psi_{2}\right)\in R\times R\times L_{F_{u}}^{2}\left(\Omega ;R\right),\lambda \in \left[0,1\right],u\in \left[s,T+K\right]$*,*
  $$\begin{array}{cc} & f\left(t,s,\lambda y_{1}+\left(1-\lambda \right)y_{2},\lambda z_{1}+\left(1-\lambda \right)z_{2},\lambda \psi_{1}+\left(1-\lambda \right)\psi_{2}\right)\\ & \leq \lambda f\left(t,s,y_{1},z_{1},\psi_{1}\right)+\left(1-\lambda \right)g\left(t,s,y_{2},z_{2},\psi_{2}\right),\;\;\;\;\;\left(t,s\right)\in ▵,\;a.s. \end{array}$$
  *Then for any $\xi_{1}\left(\cdot \right),\xi_{2}\left(\cdot \right)\in L_{F_{T}}^{2}\left(0,T+K;R\right)$ and any λ∈[0,1],*
  $$\begin{array}{cc} & \rho_{t}\left(\lambda \xi_{1}\left(\cdot \right)+\left(1-\lambda \right)\xi_{2}\left(\cdot \right)\right)\\ & \leq \lambda \rho_{t}\left(\xi_{1}\left(\cdot \right)\right)+\left(1-\lambda \right)\rho_{t}\left(\xi_{2}\left(\cdot \right)\right),\;\;\;\;a.s.,\;t\in \left[0,T+K\right]. \end{array}$$
*(ii)*  *(Cash invariance) There has a deterministic integrable function r(·) satisfying for each $\xi \left(\cdot \right)\in L_{F_{T}}^{2}\left(0,T+K;R\right)$ and each constant c,*
  $$\rho_{t}\left(\xi \left(\cdot \right)+c\right)=\rho_{t}\left(\xi \left(\cdot \right)\right)-ce^{-\int_{t}^{T}r\left(s\right)ds},\;\;\;\;a.s.,\;t\in \left[0,T+K\right].$$
*(iii)*  *(Past independence) For each $\xi_{1}\left(\cdot \right),\xi_{2}\left(\cdot \right)\in L_{F_{T}}^{2}\left(0,T+K;R\right)$, if*
  $$\xi_{1}\left(s\right)=\xi_{2}\left(s\right),\;\;\;\;a.s.,\;s\in \left[t,T+K\right]$$
  *for some t∈[0,T+K), then*
  $$\rho_{t}\left(\xi_{1}\left(\cdot \right)\right)=\rho_{t}\left(\xi_{2}\left(\cdot \right)\right),\;\;a.s.$$
*(iv)*  *(Monotonicity) For each $\xi_{1}\left(\cdot \right),\xi_{2}\left(\cdot \right)\in L_{F_{T}}^{2}\left(0,T+K;R\right)$, if*
  $$\xi_{1}\left(s\right)\leq \xi_{2}\left(s\right),\;\;\;\;a.s.,\;s\in \left[t,T+K\right],$$
  *for some t∈[0,T+K), then*
  $$\rho_{s}\left(\xi_{1}\left(\cdot \right)\right)\geq \rho_{s}\left(\xi_{2}\left(\cdot \right)\right),\;\;\;\;a.s.,\;s\in \left[t,T+K\right].$$

**Remark** **4.** *Under Theorem 3, ρ is a dynamic coherent risk measure if the following conditions hold:*

*(Subadditivity) Assume that f is subadditive in (y,z,ψ), i.e., for each*$(y_{1},z_{1},$$\psi_{1}),(y_{2},z_{2},\psi_{2})\in R\times R\times L_{F_{u}}^{2}\left(\Omega ;R\right),u\in [s,T+K],$
  $$\begin{array}{cc} \; & f(t,s,y_{1}+y_{2},z_{1}+z_{2},\psi_{1}+\psi_{2})\\ \; & \;\;\;\;\leq f\left(t,s,y_{1},z_{1},\psi_{1}\right)+f\left(t,s,y_{2},z_{2},\psi_{2}\right),\;\;\;\;\left(t,s\right)\in ▵,a.s. \end{array}$$
    *Then for each $\xi \left(\cdot \right),\eta \left(\cdot \right)\in L_{F_{T}}^{2}\left(0,T+K;R\right)$,*
  $$\rho_{t}\left(\xi \left(\cdot \right)+\eta \left(\cdot \right)\right)\leq \rho_{t}\left(\xi \left(\cdot \right)+\eta \left(\cdot \right)\right),\;\;\;\;a.s.,\;t\in \left[0,T+K\right].$$
*(Positive homogeneity) Assume that f is positively homogeneous in (y,z,ψ), and g is positively homogeneous in (y,z), i.e., for each $\left(y,z,\psi \right)\in R\times R\times L_{F_{u}}^{2}\left(\Omega ;R\right),u\in \left[s,T+K\right]$ and all α>0,*
  $$\begin{array}{cc} \; & f\left(t,s,\alpha y,\alpha z,\alpha \psi \right)=\alpha f\left(t,s,y,z,\psi \right),\\ \; & g\left(t,s,\alpha y,\alpha z\right)=\alpha g\left(t,s,y,z\right),\;\;\;\left(t,s\right)\in ▵,\;a.s. \end{array}$$
    *Then for each $\xi \left(\cdot \right)\in L_{F_{T}}^{2}\left(0,T+K;R\right)$ and all α>0,*
  $$\rho_{t}\left(\alpha \xi \left(\cdot \right)\right)=\alpha \rho_{t}\left(\xi \left(\cdot \right)\right),\;\;\;\;a.s.,\;t\in \left[0,T\right].$$

## 4. Proofs of Main Results

**Proof of Theorem** **1.** For the simplification of proof, we first use some results of theEquation (3) to prove the existence of the following ABDSVIE,

(17) $$\left\{\begin{array}{cc} Y(t) & =\xi \left(t\right)+\int_{t}^{T}f\left(t,s,Y(s+\zeta (s)),Z(t,s+\delta (s)),Z(s+\delta (s),t)\right)ds\\ \; & \;\;\;\;+\int_{t}^{T}g\left(t,s,Y\left(s\right),Z\left(t,s\right),Z\left(s,t\right)\right)d\overset{\leftarrow }{B}\left(s\right)\\ \; & \;\;\;\;-\int_{t}^{T}Z\left(t,s\right)dW\left(s\right),\;\;\;t\in \left[0,T\right];\\ Y(t) & =\xi (t),\;\;\;t\in [T,T+K];\\ Z(t,s) & =\eta \left(t,s\right),\;\;\;\left(t,s\right)\in {\left[0,T+K\right]}^{2}\backslash {\left[0,T\right]}^{2}. \end{array}\right.$$

In order to obtain the existence to (17), we consider the following simple equation for each $\left(\xi \left(\cdot \right),\eta \left(\cdot ,\cdot \right)\right)\in M^{2}\left[0,T+K\right]$,

(18) $$\left\{\begin{array}{cc} Y(t) & =\xi \left(t\right)+\int_{t}^{T}\bar{f}\left(t,s\right)ds+\int_{t}^{T}\bar{g}\left(t,s\right)d\overset{\leftarrow }{B}\left(s\right)\\ \; & \;\;\;\;-\int_{t}^{T}Z\left(t,s\right)dW\left(s\right),\;\;\;t\in \left[0,T\right];\\ Y(t) & =\xi (t),\;\;\;t\in [T,T+K];\\ Z(t,s) & =\eta \left(t,s\right),\;\;\;\left(t,s\right)\in {\left[0,T+K\right]}^{2}\backslash {\left[0,T\right]}^{2}, \end{array}\right.$$

where $\bar{f}\left(t,s\right)=f\left(t,s,Y(s+\zeta (s)),Z(t,s+\delta (s)),Z(s+\delta (s),t)\right),\bar{g}\left(t,s\right)=g\left(t,s,Y\left(s\right),Z\left(t,s\right),Z\left(s,t\right)\right)$. By Proposition 1 and 2, BDSVIE (18) has a unique solution in $H_{▵}^{2}\left[0,T\right]$, denoted by (Y(·),Z(·,·)). Suppose that Z(·,·) defined on ${▵}^{c}$ satisfies (4).

Note that for each $\left(t,s\right)\in {\left[0,T+K\right]}^{2}\backslash {\left[0,T\right]}^{2}$,

Y(t)=ξ(t),t∈[T,T+K];Z(t,s)=η(t,s),

where $\left(\xi \left(\cdot \right),\eta \left(\cdot ,\cdot \right)\right)\in M^{2}\left[0,T+K\right]$. Then, we get that BDSVIE (18) has an M-solution in $M^{2}\left[0,T+K\right]$, denoted by (Y(·),Z(·,·)).

Therefore, for each $\left(y\left(\cdot \right),z\left(\cdot ,\cdot \right)\right)\in M^{2}\left[0,T+K\right]$, define the map $\Theta :M^{2}\left[0,T+K\right]\to M^{2}\left[0,T+K\right]$ as follows:

Θ(y(·),z(·,·)):=(Y(·),Z(·,·)).

Under the norm ${∥\cdot ∥}_{M^{2}\left[0,T+K\right]}$, we now need to prove that the map Θ(·,·) is a contractive.

For all $\left(y_{1}\left(\cdot \right),z_{1}\left(\cdot ,\cdot \right)\right),\left(y_{2}\left(\cdot \right),z_{2}\left(\cdot ,\cdot \right)\right)\in M^{2}\left[0,T+K\right]$, let

$$\left(Y_{1}\left(\cdot \right),Z_{1}\left(\cdot ,\cdot \right)\right)=\Theta \left(y_{1}\left(\cdot \right),z_{1}\left(\cdot ,\cdot \right)\right),\;\;\;\left(Y_{2}\left(\cdot \right),Z_{2}\left(\cdot ,\cdot \right)\right)=\Theta \left(y_{2}\left(\cdot \right),z_{2}\left(\cdot ,\cdot \right)\right).$$

Their differences are also denoted by

$$\begin{array}{cc} & \left(\tilde{y}\left(\cdot \right),\tilde{z}\left(\cdot ,\cdot \right)\right)=\left(\left(y_{1}\left(\cdot \right)-y_{2}\left(\cdot \right)\right),\left(z_{1}\left(\cdot ,\cdot \right)-z_{2}\left(\cdot ,\cdot \right)\right)\right),\\ & \left(\tilde{Y}\left(\cdot \right),\tilde{Z}\left(\cdot ,\cdot \right)\right)=\left(\left(Y_{1}\left(\cdot \right)-Y_{2}\left(\cdot \right)\right),\left(Z_{1}\left(\cdot ,\cdot \right)-Z_{2}\left(\cdot ,\cdot \right)\right)\right). \end{array}$$

Applying the estimate (6), we have

(19) $$\begin{array}{cc} \; & E\left[\int_{0}^{T}\left(e^{\beta t}|\tilde{Y}{\left(t\right)|}^{2}+\int_{t}^{T}e^{\beta s}{\left|\tilde{Z}\left(t,s\right)\right|}^{2}ds\right)dt\right]\\ \; & \;\leq \frac{C}{\beta }E\left[\int_{0}^{T}\int_{t}^{T}e^{\beta s}|f\left(t,s,y_{1}\left(s+\zeta \left(s\right)\right),z_{1}\left(t,s+\delta \left(s\right)\right),z_{1}\left(s+\delta \left(s\right),t\right)\right)\right.\\ \; & \;-f\left(t,s,y_{2}\left(s+\zeta \left(s\right)\right),z_{2}\left(t,s+\delta \left(s\right)\right),z_{2}\left(s+\delta \left(s\right),t\right)\right){|}^{2}dsdt] \end{array}$$

(20) $$\begin{array}{cc} \; & +E\left[\int_{0}^{T}e^{\beta t}\int_{t}^{T}|g\left(t,s,y_{1}\left(s\right),z_{1}\left(t,s\right),z_{1}\left(s,t\right)\right)\right.\\ \; & \;\;\;\;-g\left(t,s,y_{2}\left(s\right),z_{2}\left(t,s\right),z_{2}\left(s,t\right)\right){|}^{2}dsdt] \end{array}$$

(21) $$\begin{array}{cc} \; & +E\left[\int_{0}^{T}\int_{t}^{T}e^{\beta s}|g\left(t,s,y_{1}\left(s\right),z_{1}\left(t,s\right),z_{1}\left(s,t\right)\right)\right.\\ \; & \;\;\;\;-g\left(t,s,y_{2}\left(s\right),z_{2}\left(t,s\right),z_{2}\left(s,t\right)\right){|}^{2}dsdt]. \end{array}$$

Next, in order to estimate the above inequality, we estimate the terms (19), (20) and (21), respectively. Taking square to (10), we get that

(22) $${\left|Y\left(t\right)\right|}^{2}={\left|E\left[Y\left(t\right)\right]\right|}^{2}+{\left(\int_{0}^{t}Z\left(t,s\right)dW\left(s\right)\right)}^{2}+2E\left[Y\left(t\right)\right]\int_{0}^{t}Z\left(t,s\right)dW\left(s\right).$$

With the help of the martingale property and the quadratic variation of Brownian motion, taking expectation to (22), we get that

(23) $$\begin{array}{cc} E\left[{\left|Y\left(t\right)\right|}^{2}\right] & =E\left[{\left|E\left[Y\left(t\right)\right]\right|}^{2}\right]+E\left[\int_{0}^{t}{\left|Z\left(t,s\right)\right|}^{2}ds\right]\\ \; & \geq E\left[\int_{0}^{t}{\left|Z\left(t,s\right)\right|}^{2}ds\right], \end{array}$$

which can imply that

(24) $$\begin{array}{cc} E\left[\int_{0}^{T+K}\int_{0}^{t}e^{\beta s}{\left|Z\left(t,s\right)\right|}^{2}dsdt\right] & \leq E\left[\int_{0}^{T+K}e^{\beta t}\int_{0}^{t}{\left|Z\left(t,s\right)\right|}^{2}dsdt\right]\\ \; & \leq E\left[\int_{0}^{T+K}e^{\beta t}{\left|Y\left(t\right)\right|}^{2}dt\right]. \end{array}$$

Using integration by parts, we get that

(25) $$\begin{array}{cc} \; & E\left[\int_{0}^{T+K}e^{\beta t}\left(\int_{t}^{T+K}{\left|Y\left(s\right)\right|}^{2}ds\right)dt\right]\\ \; & =\frac{1}{\beta }E\left[\int_{0}^{T+K}\beta e^{\beta t}\left(\int_{t}^{T+K}{\left|Y\left(s\right)\right|}^{2}ds\right)dt\right]\\ \; & =\frac{1}{\beta }E\left[e^{\beta t}\left(\int_{t}^{T+K}{\left|Y\left(s\right)\right|}^{2}ds\right){|}_{0}^{T+K}+\int_{0}^{T+K}e^{\beta t}{\left|Y\left(t\right)\right|}^{2}dt\right]\\ \; & \leq \frac{1}{\beta }E\left[\int_{0}^{T+K}e^{\beta t}{\left|Y\left(t\right)\right|}^{2}dt\right], \end{array}$$

and for each r∈[0,T+K], using integration by parts again, we deduce that

(26) $$\begin{array}{c} \int_{r}^{T+K}\beta e^{\beta s}\left(\int_{s}^{T+K}{\left|Z(t,u)\right|}^{2}du\right)ds=\left(e^{\beta s}\int_{s}^{T+K}{\left|Z(t,u)\right|}^{2}du\right){|}_{r}^{T+K}\\ +\int_{r}^{T+K}e^{\beta s}{\left|Z(t,s)\right|}^{2}ds. \end{array}$$

By taking r=t to (26), we get for each t∈[0,T],

(27) $$\begin{array}{cc} \; & E\left[\int_{0}^{T+K}\int_{t}^{T+K}e^{\beta s}{\left|Z(t,s)\right|}^{2}dsdt\right]\\ \; & =E\left[\int_{0}^{T+K}\int_{t}^{T+K}\beta e^{\beta s}\left(\int_{s}^{T+K}{\left|Z(t,u)\right|}^{2}du\right)dsdt\right]\\ \; & +E\left[\int_{0}^{T+K}e^{\beta t}\int_{t}^{T+K}{\left|Z(t,u)\right|}^{2}dudt\right], \end{array}$$

which implies that

(28) $$\begin{array}{c} E\left[\int_{0}^{T+K}e^{\beta t}\int_{t}^{T+K}{\left|Z(t,s)\right|}^{2}dsdt\right]\leq E\left[\int_{0}^{T+K}\int_{t}^{T+K}e^{\beta s}{\left|Z(t,s)\right|}^{2}dsdt\right],\\ E\left[\int_{0}^{T+K}e^{\beta t}\int_{t}^{T+K}{\left|Z(s,t)\right|}^{2}dsdt\right]\leq E\left[\int_{0}^{T+K}\int_{t}^{T+K}e^{\beta s}{\left|Z(s,t)\right|}^{2}dsdt\right]. \end{array}$$

In order to get the estimate of (19), by Lipschitz condition, (9), Fubini Theorem and then (24), we get that

(29) $$\begin{array}{cc} \; & E\left[\int_{0}^{T}\int_{t}^{T}e^{\beta s}|f\left(t,s,y_{1}\left(s+\zeta \left(s\right)\right),z_{1}\left(t,s+\delta \left(s\right)\right),z_{1}\left(s+\delta \left(s\right),t\right)\right)\right.\\ \; & \;\;\;\;\left.-f\left(t,s,y_{2}\left(s+\zeta \left(s\right)\right),z_{2}\left(t,s+\delta \left(s\right)\right),z_{2}\left(s+\delta \left(s\right),t\right)\right){|}^{2}dsdt\right]\\ \; & \leq LE\left[\int_{0}^{T}\int_{t}^{T}e^{\beta s}\left(\right|\tilde{y}{\left(s+\zeta \left(s\right)\right)|}^{2}+{\left|\tilde{z}\left(t,s+\delta \left(s\right)\right)\right|}^{2}\right.\\ \; & \;\;\;\;\left.+|\tilde{z}{\left(s+\delta \left(s\right),t\right)|}^{2})dsdt\right]\\ \; & \leq \;\Gamma LE\left[\int_{0}^{T+K}\int_{t}^{T+K}e^{\beta s}\left(|\tilde{z}{\left(t,s\right)|}^{2}+{\left|\tilde{z}\left(s,t\right)\right|}^{2}\right)dsdt\right]\\ \; & \;\;\;\;+\Gamma LTE\left[\int_{0}^{T+K}e^{\beta s}{\left|\tilde{y}\left(s\right)\right|}^{2}ds\right]\\ \; & \leq \Gamma L\left(T+1\right)E\left[\int_{0}^{T+K}e^{\beta s}{\left|\tilde{y}\left(s\right)\right|}^{2}ds\right]\;+\;\Gamma LE\left[\int_{0}^{T+K}\int_{t}^{T+K}e^{\beta s}{\left|\tilde{z}\left(t,s\right)\right|}^{2}dsdt\right]\\ \; & \;\;\;+\Gamma LE\left[\int_{0}^{T+K}\int_{0}^{t}e^{\beta t}{\left|\tilde{z}\left(t,s\right)\right|}^{2}dsdt\right]\\ \; & \leq 2\Gamma L\left(T+1\right)E\left[\int_{0}^{T+K}e^{\beta s}{\left|\tilde{y}\left(s\right)\right|}^{2}ds\right]\;+\;\Gamma LE\left[\int_{0}^{T+K}\int_{t}^{T+K}e^{\beta s}{\left|\tilde{z}\left(t,s\right)\right|}^{2}dsdt\right]. \end{array}$$

For the estimate of (21), by Lipschitz condition, Fubini Theorem and then (24), we obtain that

(30) $$\begin{array}{cc} \; & E\left[\int_{0}^{T}\int_{t}^{T}e^{\beta s}|g\left(t,s,y_{1}\left(s\right),z_{1}\left(t,s\right),z_{1}\left(s,t\right)\right).\right.\\ \; & \;\;\;\;-g\left(t,s,y_{2}\left(s\right),z_{2}\left(t,s\right),z_{2}\left(s,t\right)\right){|}^{2}dsdt]\\ \; & \leq \alpha E\left[\int_{0}^{T}\int_{t}^{T}e^{\beta s}\left(|\tilde{y}{\left(s\right)|}^{2}+|\tilde{z}{\left(t,s\right)|}^{2}+{\left|\tilde{z}\left(s,t\right)\right|}^{2}\right)dsdt\right]\\ \; & \leq \alpha TE\left[\int_{0}^{T}e^{\beta t}{\left|\tilde{y}\left(t\right)\right|}^{2}dt\right]+\alpha E\left[\int_{0}^{T}\int_{t}^{T}e^{\beta s}{\left|\tilde{z}\left(t,s\right)\right|}^{2}dsdt\right]\\ \; & \;\;\;\;+\alpha E\left[\int_{0}^{T}\int_{0}^{t}e^{\beta t}{\left|\tilde{z}\left(t,s\right)\right|}^{2}dsdt\right]\\ \; & \leq \alpha \left(T+1\right)E\left[\int_{0}^{T+K}e^{\beta s}{\left|\tilde{y}\left(s\right)\right|}^{2}ds\right]\;+\;\alpha E\left[\int_{0}^{T+K}\int_{t}^{T+K}e^{\beta s}{\left|\tilde{z}\left(t,s\right)\right|}^{2}dsdt\right]. \end{array}$$

Now, for the estimate of (20), by using Lipschitz condition, (25), (28), Fubini Theorem and then (24), we have

(31) $$\begin{array}{cc} \; & E\left[\int_{0}^{T}e^{\beta t}\int_{t}^{T}|g\left(t,s,y_{1}\left(s\right),z_{1}\left(t,s\right),z_{1}\left(s,t\right)\right)\right.\\ \; & \;\;\;\;-g\left(t,s,y_{2}\left(s\right),z_{2}\left(t,s\right),z_{2}\left(s,t\right)\right){|}^{2}dsdt]\\ \; & \leq \alpha E\left[\int_{0}^{T}e^{\beta t}\int_{t}^{T}\left(|\tilde{y}{\left(s\right)|}^{2}+|\tilde{z}{\left(t,s\right)|}^{2}+{\left|\tilde{z}\left(s,t\right)\right|}^{2}\right)dsdt\right]\\ \; & \leq \frac{\alpha }{\beta }E\left[\int_{0}^{T}e^{\beta t}{\left|\tilde{y}\left(t\right)\right|}^{2}dt\right]+\alpha E\left[\int_{0}^{T}\int_{t}^{T}e^{\beta s}{\left|\tilde{z}\left(t,s\right)\right|}^{2}dsdt\right]\\ \; & \;\;\;\;+\alpha E\left[\int_{0}^{T}\int_{t}^{T}e^{\beta s}{\left|\tilde{z}\left(s,t\right)\right|}^{2}dsdt\right]\\ \; & \leq \left(\frac{\alpha }{\beta }+\alpha \right)E\left[\int_{0}^{T+K}e^{\beta t}{\left|\tilde{y}\left(t\right)\right|}^{2}dt\right]\;+\;\alpha E\left[\int_{0}^{T+K}\int_{t}^{T+K}e^{\beta s}{\left|\tilde{z}\left(t,s\right)\right|}^{2}dsdt\right]. \end{array}$$

Therefore, combining (29), (30) and (31), we get

(32) $$\begin{array}{cc} \; & E\left[\int_{0}^{T}\left(e^{\beta t}|\tilde{Y}{\left(t\right)|}^{2}+\int_{t}^{T}e^{\beta s}{\left|\tilde{Z}\left(t,s\right)\right|}^{2}ds\right)dt\right]\\ \; & \;\;\;\;\leq \left(\frac{2C\Gamma L(T+1)+\alpha }{\beta }+\alpha \left(T+2\right)\right)E\left[\int_{0}^{T+K}e^{\beta s}{\left|\tilde{y}\left(s\right)\right|}^{2}ds\right]\\ \; & \;\;\;\;+\left(\frac{C\Gamma L}{\beta }+2\alpha \right)E\left[\int_{0}^{T+K}\int_{t}^{T+K}e^{\beta s}{\left|\tilde{z}\left(t,s\right)\right|}^{2}dsdt\right]. \end{array}$$

Note that

$$\tilde{Y}\left(t\right)=0,\;\;t\in \left[T,T+K\right],\;\;\;\;\tilde{Z}\left(t,s\right)=0,\;\;\left(t,s\right)\in {\left[0,T+K\right]}^{2}\backslash {\left[0,T\right]}^{2}.$$

Therefore, we have

(33) $$\begin{array}{cc} \; & E\left[\int_{0}^{T+K}\left(e^{\beta t}|\tilde{Y}{\left(t\right)|}^{2}+\int_{t}^{T+K}e^{\beta s}{\left|\tilde{Z}\left(t,s\right)\right|}^{2}ds\right)dt\right]\\ \; & \;\;\;\;\leq \varepsilon E\left[\int_{0}^{T+K}\left(e^{\beta t}|\tilde{y}{\left(t\right)|}^{2}+\int_{t}^{T+K}e^{\beta s}{\left|\tilde{z}\left(t,s\right)\right|}^{2}ds\right)dt\right], \end{array}$$

where $\varepsilon =\frac{2C\Gamma L(T+1)+\alpha }{\beta }+\alpha \left(T+2\right)$. Since $\alpha <\frac{1}{T+2}$, set $\beta >\frac{2C\Gamma L(T+1)+\alpha }{1-\alpha (T+2)}$, then the mapping Θ is a strictly contractive on $M^{2}\left[0,T+k\right]$. Thus, we have shown the uniqueness to anticipated BDSVIE (17).

Analogous to the proof of the unique M-solution of ABDSVIE (17), we can obtain that for any $\left(\xi \left(\cdot \right),\eta \left(\cdot ,\cdot \right)\right)\in M^{2}\left[0,T+K\right]$, ABDSVIE (8) has a unique M-solution $\left(Y\left(\cdot \right),Z\left(\cdot ,\cdot \right)\right)\in M^{2}\left[0,T+K\right]$ without substantial difficulty. □

**Proof of Corollary** **1.** Corollary 1 can be directly implied by Theorem 1 □

**Proof of Theorem** **2.** Suppose that $\bar{\xi }\left(\cdot \right)\in L_{F_{T}}^{2}\left(0,T+K;R\right)$ with

$$\xi^{1}\left(t\right)\leq \bar{\xi }\left(t\right)\leq \xi^{2}\left(t\right),\;\;\;\;\;\;a.s.,\;t\in \left[0,T+K\right].$$

Let us consider the unique solution in $L_{F}^{2}\left(0,T+K;R\right)\times L_{F}^{2}\left(▵;R\right)$, denoted by $(\bar{Y}\left(\cdot \right),$$\bar{Z}\left(\cdot ,\cdot \right))$, solving ABDSVIE:

$$\left\{\begin{array}{cc} \bar{Y}\left(t\right) & =\bar{\xi }\left(t\right)+\int_{t}^{T}\bar{f}\left(t,s,\bar{Y}\left(s\right),\bar{Z}\left(t,s\right),\bar{Y}\left(s+\zeta \left(s\right)\right)\right)ds\\ \; & \;\;\;\;+\int_{t}^{T}g\left(t,s,\bar{Y}\left(s\right),\bar{Z}\left(t,s\right)\right)d\overset{\leftarrow }{B}\left(s\right)-\int_{t}^{T}\bar{Z}\left(t,s\right)dW\left(s\right),\;\;\;t\in \left[0,T\right];\\ \bar{Y}\left(t\right) & =\bar{\xi }\left(t\right),\;\;\;t\in \left[T,T+K\right], \end{array}\right.$$

together with the solution in $L_{F}^{2}\left(0,T+K;R\right)\times L_{F}^{2}\left(▵;R\right)$, denoted by $\left({\tilde{Y}}_{1}\left(\cdot \right),\right.\left.{\tilde{Z}}_{1}\left(\cdot ,\cdot \right)\right)$, solving the following equation:

$$\left\{\begin{array}{cc} {\tilde{Y}}_{1}\left(t\right) & =\bar{\xi }\left(t\right)+\int_{t}^{T}\bar{f}\left(t,s,{\tilde{Y}}_{1}\left(s\right),{\tilde{Z}}_{1}\left(t,s\right),{\tilde{Y}}_{0}\left(s+\zeta \left(s\right)\right)\right)ds\\ \; & \;\;\;\;+\int_{t}^{T}g\left(t,s,{\tilde{Y}}_{1}\left(s\right),{\tilde{Z}}_{1}\left(t,s\right)\right)d\overset{\leftarrow }{B}\left(s\right)-\int_{t}^{T}{\tilde{Z}}_{1}\left(t,s\right)dW\left(s\right),\;\;\;t\in \left[0,T\right];\\ {\tilde{Y}}_{1}\left(t\right) & =\bar{\xi }\left(t\right),\;\;\;t\in \left[T,T+K\right], \end{array}\right.$$

where ${\tilde{Y}}_{0}\left(\cdot \right)=Y^{2}\left(\cdot \right)$. Notice that for all (t,y,z)∈[0,s]×R×R,a.s.,a.e.s∈[0,T],

$$\left\{\begin{array}{c} \bar{f}\left(t,s,y,z,{\tilde{Y}}_{0}\left(s+\zeta \left(s\right)\right)\right)\leq f^{2}\left(t,s,y,z,{\tilde{Y}}_{0}\left(s+\zeta \left(s\right)\right)\right),\\ \bar{\xi }\left(t\right)\leq \xi^{2}\left(t\right),\;\;\;\;a.s.,\;t\in \left[0,T+K\right]. \end{array}\right.$$

From Proposition 3, for each t∈[0,T+K], it follows that there has a zero measure set $\Omega_{t}^{1}$ with

$${\tilde{Y}}_{1}\left(t\right)\leq {\tilde{Y}}_{0}\left(t\right)=Y^{2}\left(t\right),\;\;\;\;\;\;\omega \in \Omega \backslash \Omega_{t}^{1},\;t\in \left[0,T+K\right].$$

Next, consider ABDSVIE:

$$\left\{\begin{array}{cc} {\tilde{Y}}_{2}\left(t\right) & =\bar{\xi }\left(t\right)+\int_{t}^{T}\bar{f}\left(t,s,{\tilde{Y}}_{2}\left(s\right),{\tilde{Z}}_{2}\left(t,s\right),{\tilde{Y}}_{1}\left(s+\zeta \left(s\right)\right)\right)ds\\ \; & \;\;\;\;+\int_{t}^{T}g\left(t,s,{\tilde{Y}}_{2}\left(s\right),{\tilde{Z}}_{2}\left(t,s\right)\right)d\overset{\leftarrow }{B}\left(s\right)-\int_{t}^{T}{\tilde{Z}}_{2}\left(t,s\right)dW\left(s\right),\;\;\;t\in \left[0,T\right];\\ {\tilde{Y}}_{2}\left(t\right) & =\bar{\xi }\left(t\right),\;\;\;t\in \left[T,T+K\right]. \end{array}\right.$$

Since $\bar{f}\left(t,s,y,z,\psi \right)$ is increasing in ψ, we obtain that for any (t,y,z)∈[0,s]×R×R,

$$\bar{f}\left(t,s,y,z,{\tilde{Y}}_{1}\left(s+\zeta \left(s\right)\right)\right)\leq \bar{f}\left(t,s,y,z,{\tilde{Y}}_{0}\left(s+\zeta \left(s\right)\right)\right),\;\;\;\;a.s.,a.e.s\in \left[0,T\right].$$

Thus, the similar result is given, i.e., for each t∈[0,T+K], there also has a zero measure set $\Omega_{t}^{2}$ with

$${\tilde{Y}}_{2}\left(t\right)\leq {\tilde{Y}}_{1}\left(t\right),\;\;\;\;\;\;\omega \in \Omega \backslash \Omega_{t}^{2},\;t\in \left[0,T+K\right].$$

Therefore, such a sequence ${\left\{\left({\tilde{Y}}_{n}\left(\cdot \right),{\tilde{Z}}_{n}\left(\cdot ,\cdot \right)\right)\right\}}_{n\geq 1}\in L_{F}^{2}\left(0,T+K;R\right)\times L_{F}^{2}\left(▵;R\right)$ with a zero measure set $\Omega_{t}^{n}$ can be obtained, and we have that

$$\left\{\begin{array}{cc} {\tilde{Y}}_{n}\left(t\right) & =\bar{\xi }\left(t\right)+\int_{t}^{T}\bar{f}\left(t,s,{\tilde{Y}}_{n}\left(s\right),{\tilde{Z}}_{n}\left(t,s\right),{\tilde{Y}}_{n-1}\left(s+\zeta \left(s\right)\right)\right)ds\\ \; & \;\;\;\;+\int_{t}^{T}g\left(t,s,{\tilde{Y}}_{n}\left(s\right),{\tilde{Z}}_{n}\left(t,s\right)\right)d\overset{\leftarrow }{B}\left(s\right)-\int_{t}^{T}{\tilde{Z}}_{n}\left(t,s\right)dW\left(s\right),\;\;\;t\in \left[0,T\right];\\ {\tilde{Y}}_{n}\left(t\right) & =\bar{\xi }\left(t\right),\;\;\;t\in \left[T,T+K\right]. \end{array}\right.$$

and

$$Y^{2}\left(t\right)={\tilde{Y}}_{0}\left(t\right)\geq {\tilde{Y}}_{1}\left(t\right)\geq {\tilde{Y}}_{2}\left(t\right)\cdot \cdot \cdot ,\;\;\;\;\;\;\omega \in \Omega \backslash \left(\cup_{n\geq 1}\Omega_{t}^{n}\right),\;t\in \left[0,T+K\right].$$

Now, we show that the sequence ${\left\{\left({\tilde{Y}}_{n}\left(\cdot \right),{\tilde{Z}}_{n}\left(\cdot ,\cdot \right)\right)\right\}}_{n\geq 1}$ is a Cauchy sequence in $L_{F}^{2}\left(0,T+K;R\right)\times L_{F}^{2}\left(▵;R\right).$

Using the estimate (6) and the similar approach to obtain (32) (i.e., using Lipschitz condition, (9), (25) and (28), Fubini Theorem and then (24)), we obtain that

$$\begin{array}{cc} & E\left[\int_{0}^{T}\left(e^{\beta t}{\left|{\tilde{Y}}_{n}\left(t\right)-{\tilde{Y}}_{m}\left(t\right)\right|}^{2}+\int_{t}^{T}e^{\beta s}{\left|{\tilde{Z}}_{n}\left(t,s\right)-{\tilde{Z}}_{m}\left(t,s\right)\right|}^{2}ds\right)dt\right]\\ & \leq \frac{C}{\beta }E\left[\int_{0}^{T}\int_{t}^{T}e^{\beta s}|\bar{f}\left(t,s,{\tilde{Y}}_{n}\left(s\right),{\tilde{Z}}_{n}\left(t,s\right),{\tilde{Y}}_{n-1}\left(s+\zeta \left(s\right)\right)\right)\right.\\ & \;\;\;\;\left.-\bar{f}\left(t,s,{\tilde{Y}}_{m}\left(s\right),{\tilde{Z}}_{m}\left(t,s\right),{\tilde{Y}}_{m-1}\left(s+\zeta \left(s\right)\right)\right){|}^{2}dsdt\right]\\ & \;\;\;\;+E\left[\int_{0}^{T}e^{\beta t}\int_{t}^{T}|g\left(t,s,Y_{n}\left(s\right),Z_{n}\left(t,s\right)\right)-g\left(t,s,Y_{m}\left(s\right),Z_{m}\left(t,s\right)\right){|}^{2}dsdt\right]\\ & \;\;\;\;+E\left[\int_{0}^{T}\int_{t}^{T}e^{\beta s}|g\left(t,s,Y_{n}\left(s\right),Z_{n}\left(t,s\right)\right)-g\left(t,s,Y_{m}\left(s\right),Z_{m}\left(t,s\right)\right){|}^{2}dsdt\right].\\ & \leq \varepsilon E\left[\int_{0}^{T}\left(e^{\beta t}{\left|{\tilde{Y}}_{n}\left(t\right)-{\tilde{Y}}_{m}\left(t\right)\right|}^{2}+\int_{t}^{T}e^{\beta s}{\left|{\tilde{Z}}_{n}\left(t,s\right)-{\tilde{Z}}_{m}\left(t,s\right)\right|}^{2}ds\right)dt\right]\\ & \;\;\;\;+\varepsilon E\left[\int_{0}^{T+K}e^{\beta s}{\left|{\tilde{Y}}_{n-1}\left(t\right)-{\tilde{Y}}_{m-1}t)\right|}^{2}ds\right], \end{array}$$

where $\varepsilon :=\frac{2C\Gamma L(T+1)+\alpha }{\beta }+\alpha \left(T+2\right)$. Since α(T+2)<1 and

$${\tilde{Y}}_{n}\left(t\right)-{\tilde{Y}}_{m}\left(t\right)=0,\;\;\;t\in \left[T,T+K\right].$$

By choosing $\beta =\frac{8C\Gamma L(T+1)+4\alpha }{1-3\alpha (T+2)}$, we have

$$\begin{array}{cc} & E\left[\int_{0}^{T+K}e^{\beta t}{\left|{\tilde{Y}}_{n}\left(t\right)-{\tilde{Y}}_{m}\left(t\right)\right|}^{2}dt+\int_{0}^{T}\int_{t}^{T}e^{\beta s}{\left|{\tilde{Z}}_{n}\left(t,s\right)-{\tilde{Z}}_{m}\left(t,s\right)\right|}^{2}dsdt\right]\\ & \leq \frac{\lambda }{1-\lambda }E\left[\int_{t}^{T+K}e^{\beta t}{\left|{\tilde{Y}}_{n-1}\left(t\right)-{\tilde{Y}}_{m-1}\left(t\right)\right|}^{2}dt\right], \end{array}$$

where $\lambda =\frac{1+\alpha (T+2)}{4}<\frac{1}{2}$. Thus, we obtain that ${\left\{\left({\tilde{Y}}_{n}\left(\cdot \right),{\tilde{Z}}_{n}\left(\cdot ,\cdot \right)\right)\right\}}_{n\geq 2}$ is a Cauchy sequence in $L_{F}^{2}\left(0,T+K;R\right)\times L_{F}^{2}\left(▵;R\right)$. Let $\left(\tilde{Y}\left(\cdot \right),\tilde{Z}\left(\cdot ,\cdot \right)\right)$ denote their limits, then $\left(\tilde{Y}\left(\cdot \right),\tilde{Z}\left(\cdot ,\cdot \right)\right)\in L_{F}^{2}\left(0,T+K;R\right)\times L_{F}^{2}\left(▵;R\right)$ such that

$$\lim_{n\to \infty }E\left[\int_{0}^{T+K}e^{\beta t}{\left|{\tilde{Y}}_{n}\left(t\right)-\tilde{Y}\left(t\right)\right|}^{2}dt+\int_{0}^{T}\int_{t}^{T}e^{\beta s}{\left|{\tilde{Z}}_{n}\left(t,s\right)-\tilde{Z}\left(t,s\right)\right|}^{2}dsdt\right]=0,$$

Furthermore, we get that

$$\left\{\begin{array}{cc} \tilde{Y}\left(t\right) & =\bar{\xi }\left(t\right)+\int_{t}^{T}\bar{f}\left(t,s,\tilde{Y}\left(s\right),\tilde{Z}\left(t,s\right),\tilde{Y}\left(s+\zeta \left(s\right)\right)\right)ds\\ \; & \;\;\;\;+\int_{t}^{T}g\left(t,s,\tilde{Y}\left(s\right),\tilde{Z}\left(t,s\right)\right)d\overset{\leftarrow }{B}\left(s\right)-\int_{t}^{T}\tilde{Z}\left(t,s\right)dW\left(s\right),\;\;\;t\in \left[0,T\right];\\ \tilde{Y}\left(t\right) & =\bar{\xi }\left(t\right),\;\;\;t\in \left[T,T+K\right]. \end{array}\right.$$

From the uniqueness to ABDSVIE, it follows that

$$\bar{Y}\left(t\right)=\tilde{Y}\left(t\right)\leq {\tilde{Y}}_{0}\left(t\right)=Y^{2}\left(t\right),\;\;\;\;\;\;\;a.s.,\;t\in \left[0,T+K\right].$$

The similar argument is also given that

$$Y^{1}\left(t\right)\leq \bar{Y}\left(t\right),\;\;\;\;\;\;\;a.s.,t\in \left[0,T+K\right].$$

Thus, the result has been completed. □

**Proof of Theorem** **3.** (Monotonicity) Let $\xi_{1}\left(\cdot \right)\leq \xi_{2}\left(\cdot \right)$ satisfy $\xi_{1}\left(\cdot \right),\xi_{2}\left(\cdot \right)\in L_{F_{T}}^{2}\left(0,T\;+\;K;R\right)$. By Theorem 2, it is easy to know that

$$\rho_{t}\left(\xi_{1}\left(\cdot \right)\right)\geq \rho_{t}\left(\xi_{2}\left(\cdot \right)\right),\;\;\;t\in \left[t,T+K\right]$$

(Past independence) The definition of ρ can directly imply the past independence.

(Cash invariance) Notice that $f\left(t,s,y,z,\psi \right)=r\left(s\right)\left(y+\psi \right)+f_{0}\left(t,s,z\right)$. Let $\xi \left(\cdot \right)\in L_{F_{T}}^{2}\left(0,T+K;R\right)$ be fixed and let $\left(Y^{c}\left(\cdot \right),Z^{c}\left(\cdot ,\cdot \right)\right)$ be the solution of (15) corresponding ξ(·)+c for any c∈R. We have to show that

$$Y^{c}\left(t\right)=Y^{0}\left(t\right)-ce^{-\int_{t}^{T}r\left(u\right)du}.$$

In fact,

(34) $$\begin{array}{c} -\xi \left(t\right)\;-\;c+\int_{t}^{T}\left[r\left(s\right)\left(Y^{0}\left(s\right)\;+\;Y^{0}\left(s\;+\;\zeta \left(s\right)\right)\;-\;ce^{-\int_{t}^{T}r\left(u\right)du}\right)\;+\;f_{0}\left(t,s,Z^{0}\left(t,s\right)\right)\right]ds\\ +\int_{t}^{T}g\left(t,s,Z^{0}\left(t,s\right)\right)d\overset{\leftarrow }{B}\left(s\right)-\int_{t}^{T}Z^{0}\left(t,s\right)dW\left(s\right)\\ =Y^{0}\left(t\right)-c+c\int_{t}^{T}r\left(s\right)e^{-\int_{t}^{T}r\left(u\right)du}ds\\ =Y^{0}\left(t\right)-c+ce^{-\int_{t}^{T}r\left(u\right)du}{|}_{t}^{T}=Y^{0}\left(t\right)-ce^{-\int_{t}^{T}r\left(s\right)ds}. \end{array}$$

By the uniqueness to ABDSVIE, we can obtain that for any t∈[t,T],

$$\begin{array}{c} Y^{c}\left(t\right)=Y^{0}\left(t\right)-ce^{-\int_{t}^{T}r\left(u\right)du},\;\;\;Z^{c}\left(t,s\right)=Z^{0}\left(t,s\right). \end{array}$$

When Y(t)=−ξ(t),t∈[T,T+K]. By choosing r(s)=0,s∈[T,T+K], we have

$$\begin{array}{c} Y^{c}\left(t\right)=Y^{0}\left(t\right)-ce^{-\int_{t}^{T}r\left(u\right)du},\;\;\;t\in \left[0,T+K\right]. \end{array}$$

(Convexity) Let $\left(Y^{1}\left(\cdot \right),Z^{1}\left(\cdot ,\cdot \right)\right)$, $\left(Y^{2}\left(\cdot \right),Z^{2}\left(\cdot ,\cdot \right)\right)$ and $\left(\tilde{Y}\left(\cdot \right),\tilde{Z}\left(\cdot ,\cdot \right)\right)$ denote the solutions of ABDSVIE (15) corresponding to the terminal conditions $\xi_{1}\left(\cdot \right)$, $\xi_{2}\left(\cdot \right)$ and $\lambda \xi_{1}\left(\cdot \right)+\left(1-\lambda \right)\xi_{2}\left(\cdot \right)$, respectively. Then we have

$$\left\{\begin{array}{cc} \tilde{Y}\left(t\right) & =-\lambda \xi_{1}\left(\cdot \right)-\left(1-\lambda \right)\xi_{2}\left(\cdot \right)+\int_{t}^{T}f\left(t,s,\tilde{Y}\left(s\right),\tilde{Z}\left(t,s\right),\tilde{Y}\left(s+\delta \left(s\right)\right)\right)ds\\ \; & \;\;\;\;+\int_{t}^{T}g\left(t,s,\tilde{Z}\left(t,s\right)\right)d\overset{\leftarrow }{B}\left(s\right)-\int_{t}^{T}\tilde{Z}\left(t,s\right)dW\left(s\right),\;\;\;t\in \left[0,T\right];\\ \tilde{Y}\left(t\right) & =-\lambda \xi_{1}\left(\cdot \right)-\left(1-\lambda \right)\xi_{2}\left(\cdot \right),\;\;\;t\in \left[T,T+K\right]. \end{array}\right.$$

We have to show that for all λ∈[0,1] and $\xi_{1}\left(\cdot \right),\xi_{2}\left(\cdot \right)\in L_{F_{T}}^{2}\left(0,T+K;R\right)$,

$$\rho_{t}\left(\lambda \xi_{1}\left(\cdot \right)+\left(1-\lambda \right)\xi_{2}\left(\cdot \right)\right)\leq \lambda \rho_{t}\left(\xi_{1}\left(\cdot \right)\right)+\left(1-\lambda \right)\rho_{t}\left(\xi_{2}\left(\cdot \right)\right),\;\;\;\;a.s.,\;t\in \left[0,T+K\right],$$

that is, for any $\xi_{1}\left(\cdot \right),\xi_{2}\left(\cdot \right)\in L_{F_{T}}^{2}\left(0,T+K;R\right),\lambda \in \left[0,1\right]$,

$$\tilde{Y}\left(t\right)\leq \lambda Y^{1}\left(t\right)+\left(1-\lambda \right)Y^{2}\left(t\right),\;\;\;\;\;\;\;\;t\in \left[0,T+K\right].$$

For i=1,2, we consider the following ABDSVIEs:

(35) $$\left\{\begin{array}{cc} Y^{i}\left(t\right) & =-\xi^{i}\left(t\right)+\int_{t}^{T}f\left(t,s,Y^{i}\left(s\right),Z^{i}\left(t,s\right),Y^{i}\left(s+\zeta \left(s\right)\right)\right)ds\\ \; & \;\;\;\;+\int_{t}^{T}g\left(t,s,Z^{i}\left(t,s\right)\right)d\overset{\leftarrow }{B}\left(s\right)-\int_{t}^{T}Z^{i}\left(t,s\right)dW\left(s\right),\;\;\;t\in \left[0,T\right];\\ Y^{i}\left(t\right) & =-\xi^{i}\left(t\right),\;\;\;t\in \left[T,T+K\right]. \end{array}\right.$$

We set for any λ∈[0,1],

$$X=\lambda \xi_{1}\left(\cdot \right)+\left(1-\lambda \right)\xi_{2}\left(\cdot \right),\;\bar{Y}=\lambda Y^{1}+\left(1-\lambda \right)Y^{2},\;\bar{Z}=\lambda Z^{1}+\left(1-\lambda \right)Z^{2}.$$

Note that *f* is convex in (y,z,ψ). Thus, for all (t,s)∈▵, we have that

$$\begin{array}{cc} \; & \lambda Y^{1}\left(t\right)+\left(1-\lambda \right)Y^{2}\left(t\right)\\ \; & =-\lambda \xi_{1}\left(t\right)-\left(1-\lambda \right)\xi_{2}\left(t\right)+\int_{t}^{T}\lambda f\left(t,s,Y^{1}\left(s\right),Z^{1}\left(t,s\right),Y^{1}\left(s+\zeta \left(s\right)\right)\right)ds\\ \; & \;\;\;\;+\int_{t}^{T}\left(1-\lambda \right)f\left(t,s,Y^{2}\left(s\right),Z^{2}\left(t,s\right),Y^{2}\left(s+\zeta \left(s\right)\right)\right)ds\\ \; & \;\;\;\;+\int_{t}^{T}\lambda g\left(t,s,Z^{1}\left(t,s\right)\right)d\overset{\leftarrow }{B}\left(s\right)+\int_{t}^{T}\left(1-\lambda \right)g\left(t,s,Z^{2}\left(t,s\right)\right)d\overset{\leftarrow }{B}\left(s\right)\\ \; & \;\;\;\;-\int_{t}^{T}\left(\lambda Z^{1}\left(t,s\right)+\left(1-\lambda \right)Z^{2}\left(t,s\right)\right)dW\left(s\right)\\ \; & =-X\;+\;\int_{t}^{T}\lambda f\left(t,s,Y^{1}\left(s\right),Z^{1}\left(t,s\right),Y^{1}\left(s+\zeta \left(s\right)\right)\right)\\ \; & \;\;\;\;+\left(1\;-\;\lambda \right)f\left(t,s,Y^{2}\left(s\right),Z^{2}\left(t,s\right),Y^{1}\left(s+\zeta \left(s\right)\right)\right)ds\\ \; & \;\;\;\;+\int_{t}^{T}\lambda g\left(t,s,Z^{1}\left(t,s\right))\right)+\left(1-\lambda \right)g\left(t,s,Z^{2}\left(t,s\right)\right)d\overset{\leftarrow }{B}\left(s\right)\\ \; & \;\;\;\;-\int_{t}^{T}\bar{Z}\left(t,s\right)dW\left(s\right)\\ \; & \geq -X+\int_{t}^{T}f\left(t,s,\bar{Y}\left(s\right),\bar{Z}\left(t,s\right),\bar{Y}(s+\zeta \left(s\right)\right)ds\\ \; & \;\;\;\;+\int_{t}^{T}\lambda g\left(t,s,Z^{1}\left(t,s\right)\right)+\left(1-\lambda \right)g\left(t,s,Z^{2}\left(t,s\right)\right)d\overset{\leftarrow }{B}\left(s\right)\\ \; & \;\;\;\;-\int_{t}^{T}\bar{Z}\left(t,s\right)dW\left(s\right). \end{array}$$

Since $X=\lambda \xi_{1}\left(\cdot \right)+\left(1-\lambda \right)\xi_{2}\left(\cdot \right)$, by the Comparison theorem 2, we get that for all $\lambda \in \left[0,1\right],\xi_{1}\left(\cdot \right),\xi_{2}\left(\cdot \right)\in L_{F_{T}}^{2}\left(0,T+K;R\right)$,

$$\tilde{Y}\left(t\right)\leq \lambda Y^{1}\left(t\right)+\left(1-\lambda \right)Y^{2}\left(t\right),\;\;\;\;\;\;\;\;t\in \left[0,T\right].$$

Note that $Y^{i}\left(t\right)=-\xi^{i}\left(t\right),i=1,2,t\in \left[T,T+K\right]$. Thus, we have

$$\tilde{Y}\left(t\right)\leq \lambda Y^{1}\left(t\right)+\left(1-\lambda \right)Y^{2}\left(t\right),\;\;\;\;\;\;\;\;t\in \left[0,T+K\right].$$

We have completed the proof. □

## 5. Conclusions

In this paper, based on the consideration of the existence of some inside and future market information in the financial market, a class of ABDSVIEs are introduced and used to induce dynamic risk measures for risk quantification. The theory, including the existence, uniqueness and a comparison theorem for ABDSVIEs, is provided. Finally, dynamic risk measures by ABDSVIEs are presented. Now, we are not sure whether ABDSVIEs can be used to optimize the multifactor vintage capital model of [25,26,27]. It is a very interesting and meaningful research topic for our future work.
